# Cardiovascular Disease in Pregnancy: When Two Hearts Beat as One

**DOI:** 10.3390/diagnostics15222921

**Published:** 2025-11-19

**Authors:** Chiara Tognola, Filippo Brucato, Alessandro Maloberti, Marisa Varrenti, Alberto Preda, Patrizio Mazzone, Cristina Giannattasio, Fabrizio Guarracini

**Affiliations:** 1 Clinical Cardiology Unit, De Gasperis Cardio Center, Niguarda Hospital, 20162 Milan, Italy; alessandro.maloberti@ospedaleniguarda.it (A.M.); cristina.giannattasio@ospedaleniguarda.it (C.G.); 2 School of Medicine and Surgery, University of Milano-Bicocca, 20126 Milan, Italy; f.brucato@campus.unimib.it; 3 Electrophysiology Unit, De Gasperis Cardio Center, Niguarda Hospital, 20162 Milan, Italy; marisa.varrenti@ospedaleniguarda.it (M.V.); alberto.preda@ospedaleniguarda.it (A.P.); patrizio.mazzone@ospedaleniguarda.it (P.M.); fabrizio.guarracini@ospedaleniguarda.it (F.G.)

**Keywords:** pregnancy, cardiovascular disease, peripartum cardiomyopathy, hypertensive disorders, valvular disease, anticoagulation, endothelial dysfunction, pregnancy heart team, postpartum follow-up

## Abstract

**Background**: Cardiovascular disease (CVD) in pregnancy is a major cause of maternal morbidity and mortality, accounting for nearly one-third of pregnancy-related deaths worldwide. Physiological adaptations—expanded plasma volume, increased cardiac output, and a prothrombotic state—represent a natural cardiovascular stress test that may precipitate decompensation or unmask subclinical disease. **Aim**: This review critically examines contemporary evidence and international guidelines on the management of pregnancy-related cardiovascular disorders, focusing on pathophysiological mechanisms, diagnostic challenges, and therapeutic controversies. **Content**: The discussion centers on three high-impact clinical domains: (1) peripartum and preexisting cardiomyopathies, emphasizing mechanisms, prognosis, and the role of bromocriptine; (2) anticoagulation management in women with mechanical prosthetic valves, balancing maternal safety and fetal protection; and (3) hypertensive disorders of pregnancy, highlighting recent evidence from the CHAP and WILL trials and their implications for long-term cardiovascular prevention. Comparative analysis of ESC 2025 and AHA 2020 recommendations reveals broad consensus but persistent discrepancies in anticoagulation targets, postpartum surveillance, and follow-up strategies. **Perspectives**: Endothelial dysfunction, angiogenic imbalance, and systemic inflammation emerge as shared mechanisms linking diverse pregnancy-related cardiovascular conditions. Strengthening multidisciplinary care through Pregnancy Heart Teams, integrating obstetric and cardiologic expertise, and establishing structured postpartum follow-up pathways are essential to improve outcomes.

## 1. Introduction

Cardiovascular disease (CVD) in pregnancy is a major cause of maternal morbidity and mortality, accounting for nearly one-third of pregnancy-related deaths worldwide [1,2,3]. Its rising prevalence reflects the growing number of women with congenital heart disease, cardiomyopathies, valvular lesions, and arrhythmias who reach childbearing age, as well as increased maternal age and cardiometabolic risk [1,2]. Pregnancy represents a physiological cardiovascular stress test: the substantial hemodynamic, hormonal, and hematologic adaptations—expanded plasma volume, increased cardiac output, and a prothrombotic state—can unmask latent dysfunction or precipitate decompensation in women with underlying disease [3].

Beyond acute complications, adverse pregnancy outcomes (APOs) such as pre-eclampsia, gestational hypertension, and preterm delivery have emerged as female-specific cardiovascular risk enhancers. Women with a history of APOs face a two- to four-fold increased lifetime risk of chronic hypertension, ischemic heart disease, and stroke [4,5,6,7]. Thus, pregnancy and postpartum care should extend beyond obstetric outcomes to encompass long-term cardiovascular prevention.

Recent international guidelines have emphasized the role of multidisciplinary management through dedicated Pregnancy Heart Teams, integrating cardiology, obstetrics, anesthesia, and neonatology [1,8,9]. The 2025 ESC guidelines promote structured, lifelong care pathways—from preconception counseling to postpartum follow-up—to improve maternal and fetal outcomes. However, important differences persist between the ESC (2025) and AHA (2020) recommendations regarding anticoagulation strategies, antihypertensive thresholds, and delivery timing [1,2].

Risk stratification tools such as the modified WHO (mWHO) classification and the CARPREG II score remain essential but incomplete, as they mainly predict cardiac complications and underrepresent obstetric and social factors [8,9,10]. Refining these models and validating them across diverse populations are key future priorities.

Given the breadth of cardiovascular conditions in pregnancy, this review concentrates on three clinically and pathophysiologically significant topics where evidence remains limited and management controversial:

- Peripartum and pre-existing cardiomyopathies, focusing on mechanisms, prognosis, and evolving therapeutic strategies;
- Anticoagulation and prosthetic valve management, balancing maternal thrombotic protection and fetal safety;
- Hypertensive disorders of pregnancy, paradigmatic of endothelial and metabolic dysfunction linking obstetric and long-term cardiovascular disease.

By critically comparing ESC and AHA recommendations and synthesizing current pathophysiological insights, this review aims to offer practical, evidence-based guidance and highlight persistent knowledge gaps in the cardiovascular care of pregnant women.

## 2. Peripartum and Preexisting Cardiomyopathies

### 2.1. Pathophysiology and Mechanistic Insights

Heart failure (HF) is among the most serious cardiovascular complications in pregnancy, responsible for a substantial share of maternal morbidity and mortality [1,2]. The hemodynamic stress of gestation—volume expansion, increased cardiac output, and reduced afterload—can unmask latent ventricular dysfunction or exacerbate pre-existing disease. Women with impaired left-ventricular ejection fraction (LVEF), restrictive physiology, or previous decompensation are at particular risk [11].

Peripartum cardiomyopathy (PPCM) represents the most distinctive pregnancy-related cardiomyopathy. It is defined as HF with LVEF < 45% developing in late pregnancy or within several months postpartum, in the absence of another identifiable cause [12]. Incidence varies widely—from approximately 1 in 2000–4000 deliveries in Europe and North America to 1 in 300 in some African regions [12,13]. Risk factors include multiparity, African ancestry, hypertensive disorders, and advanced maternal age [1,13].

The pathophysiology of PPCM is multifactorial, involving interaction between vascular, inflammatory, and metabolic stress pathways. Key mechanisms include oxidative stress leading to cleavage of the nursing hormone prolactin into a 16 kDa fragment with anti-angiogenic and pro-apoptotic effects on endothelium and myocardium [14]. These findings have therapeutic implications. Inhibition of prolactin secretion with bromocriptine may counteract the toxic cleavage process and promote recovery. The EORP Peripartum Cardiomyopathy Registry confirmed higher LV recovery and lower six-month mortality in women receiving bromocriptine in addition to standard HF therapy, though thromboembolic complications necessitate concurrent anticoagulation [15]. Randomized trials remain limited, but mechanistic rationale and real-world data support its use in selected patients [15,16].

**Figure 1:**
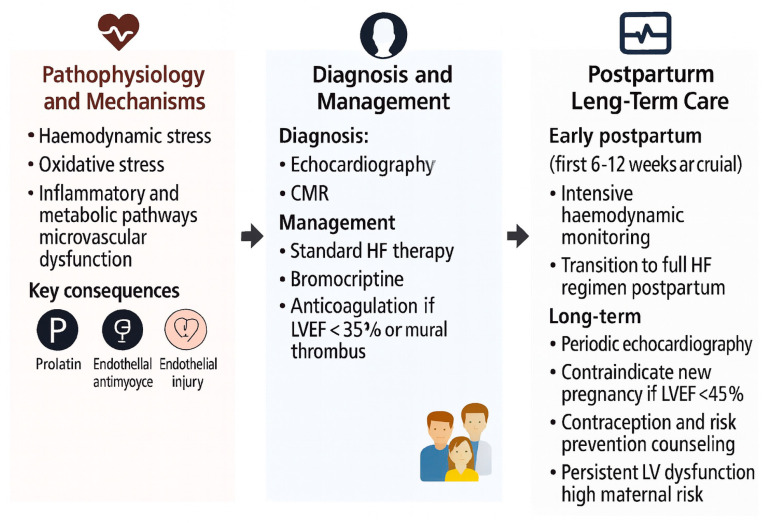
Pathophysiology, management, and prognosis of peripartum and preexisting cardiomyopathies. Abbreviations: ACEi, angiotensin-converting enzyme inhibitor; ARB, angiotensin receptor blocker; BNP, B-type natriuretic peptide; CMR, cardiovascular magnetic resonance; HF, heart failure; LMWH, low-molecular-weight heparin; LVEF, left ventricular ejection fraction; LV, left ventricle; NT-proBNP, N-terminal pro-B-type natriuretic peptide; PAH, pulmonary arterial hypertension; PPCM, peripartum cardiomyopathy.

The complex pathophysiological mechanisms underlying peripartum cardiomyopathy—including hormonal activation, oxidative stress, and the anti-angiogenic 16 kDa prolactin fragment—are summarized in Figure 1.

### 2.2. Diagnosis and Risk Stratification

Diagnosis of PPCM is often delayed because early symptoms—fatigue, dyspnea, or edema—mimic normal pregnancy. Echocardiography remains the cornerstone for assessment of systolic function and chamber size. CMR can refine diagnosis by excluding myocarditis or ischemia, although gadolinium use is limited to postpartum evaluation. Natriuretic peptides are typically elevated but may overlap with physiologic increases in late gestation [1].

Prognosis is heterogeneous. Approximately half of patients recover normal LV function within six months, whereas persistent systolic dysfunction portends adverse outcomes and high recurrence risk in subsequent pregnancies [17]. Poor prognostic markers include LVEF < 30% at diagnosis, LV end-diastolic dimension > 60 mm, and delayed presentation [18].

Recent evidence from the Italian Multicentre Registry further highlights that clinical and echocardiographic predictors of left ventricular recovery in PPCM remain insufficiently defined. In this large contemporary cohort, baseline echocardiographic findings exhibited substantial heterogeneity, and no individual parameter reliably predicted systolic function normalization. These findings underscore the need for dedicated prospective studies to refine prognostic assessment in PPCM [19].

Traditional HF scores are not validated for PPCM. The 2025 ESC guidelines recommend early risk stratification using LVEF and right-ventricular function, integration of biomarkers where available, and multidisciplinary follow-up through a Pregnancy Heart Team [1].

Emerging data also indicate that a subset of patients may present with an arrhythmic phenotype of PPCM. Recent case series have described initial manifestations dominated by malignant ventricular arrhythmias, including cardiac arrest, which may precede overt ventricular dysfunction. This arrhythmic presentation warrants particular attention, as it may alter the diagnostic pathway, risk stratification, and early management of affected women [20].

### 2.3. Management and Evidence Gaps

Treatment requires balancing maternal stabilization with fetal safety. Diuretics may relieve congestion but should be titrated to avoid reduced placental perfusion. Beta-blockers improve outcomes in systolic dysfunction; metoprolol or labetalol are preferred, whereas atenolol is avoided due to fetal growth restriction [16]. Vasodilators such as hydralazine and nitrates are safe alternatives when afterload reduction is needed; ACE inhibitors, ARBs, and sacubitril/valsartan are contraindicated until after delivery [1]. Anticoagulation is indicated when LVEF < 35% or with mural thrombus, due to heightened thrombotic risk [21,22]. Bromocriptine, administered at 2.5 mg twice daily for two weeks followed by 2.5 mg daily for six weeks with prophylactic LMWH, is endorsed by ESC 2025 for severe PPCM (Class IIa recommendation) [1].

The AHA 2020 statement is more conservative, supporting bromocriptine use only in research or specialized settings [2].

During delivery, hemodynamic shifts may precipitate decompensation; vaginal delivery with regional anesthesia and assisted second stage is preferred unless obstetric or cardiac deterioration mandates cesarean section [1]. Postpartum, introduction of guideline-directed HF therapy (ACE inhibitors/ARBs, beta-blockers, mineralocorticoid receptor antagonists) is crucial once breastfeeding and safety considerations are addressed.

### 2.4. Other Preexisting Cardiomyopathies

Beyond PPMC, pregnancy may exacerbate or unmask other pre-existing cardiomyopathies. In women with dilated or restrictive cardiomyopathy, the physiological increase in plasma volume and cardiac output imposes a substantial hemodynamic load that can precipitate heart failure, particularly when left ventricular systolic function is already impaired. The recurrence risk of decompensation in subsequent pregnancies is considerable, approaching 50% when the LVEF is below 45% prior to conception.

In hypertrophic obstructive cardiomyopathy (HOCM), the tachycardia and reduced preload characteristic of pregnancy can amplify the dynamic left ventricular outflow tract gradient. A resting or provocable gradient exceeding 50 mmHg identifies women at higher risk of syncope, arrhythmic events, or hemodynamic deterioration. Beta-blockers remain the first-line therapy to control heart rate and reduce obstruction [23].

Another complex situation involves arrhythmogenic right ventricular cardiomyopathy, in which hormonal and hemodynamic fluctuations during pregnancy may increase susceptibility to ventricular arrhythmias. Continuous rhythm monitoring and individualized adjustment of antiarrhythmic therapy are recommended, especially in the peripartum period [24]. According to the 2025 ESC guidelines, pre-pregnancy counseling is essential for all women with known cardiomyopathy, ensuring optimization of medical therapy and delivery planning within tertiary care centers equipped with multidisciplinary teams [1].

### 2.5. Postpartum and Long-Term Care

The postpartum phase represents a period of maximal risk. Venous return and systemic vascular resistance rise abruptly, increasing preload and afterload. PPCM commonly manifests or worsens within weeks after delivery, when oxidative and hormonal changes peak [17,18,19]. Intensive monitoring for at least 6–12 weeks is recommended, particularly for those with residual LV dysfunction or pulmonary arterial hypertension (PAH) [1,2].

Long-term follow-up is essential. Persistent LV dysfunction carries high mortality and contraindicates future pregnancy. Even women with full recovery should undergo periodic echocardiographic surveillance, given reports of late relapse. Comprehensive counseling on contraception and cardiovascular risk prevention is advised [1,17,18].

## 3. Anticoagulation and Mechanical Valve Management

### 3.1. Pathophysiological Basis

Pregnancy is characterized by a physiological hypercoagulable state, a key adaptation that minimizes postpartum hemorrhage but substantially increases thromboembolic risk [21,22]. This prothrombotic shift results from elevated concentrations of fibrinogen and clotting factors VII, VIII, X, and von Willebrand factor, along with decreased protein S and enhanced platelet reactivity. Venous stasis from uterine compression and hormonal effects on the vascular endothelium further promote thrombosis.

For women with mechanical prosthetic valves, atrial fibrillation, prior venous thromboembolism (VTE), or thrombophilia, these physiological changes pose a major management challenge. Pregnancy augments both maternal thrombotic risk and fetal vulnerability to anticoagulant-related complications. Balancing effective valve protection with fetal safety therefore remains one of the most difficult aspects of maternal cardiology.

### 3.2. Evidence and Clinical Outcomes

Mechanical prosthetic valves confer the highest maternal risk among valvular disorders, with valve thrombosis rates reported between 4% and 17% depending on the anticoagulation regimen [25]. Mortality associated with valve thrombosis may approach 30% in smaller series, underscoring the critical importance of optimal anticoagulant strategy.

Vitamin K antagonists (VKAs) provide the most consistent valve protection but cross the placenta and are teratogenic, especially between 6 and 12 weeks of gestation. Fetal malformations and hemorrhage risk increase sharply with warfarin doses above 5 mg daily, whereas lower doses appear safer [26]. Conversely, low-molecular-weight heparin (LMWH) and unfractionated heparin (UFH) do not cross the placenta, thereby protecting the fetus but carrying a higher risk of maternal valve thrombosis if dosing and anti-Xa monitoring are suboptimal.

Recent registry evidence has clarified these trade-offs. The ROPAC III Registry (613 pregnancies: 411 mechanical, 202 bioprosthetic) showed a live-birth rate of 54% for mechanical versus 79% for bioprosthetic valves. Valve thrombosis occurred more often with LMWH-based regimens, especially where anti-Xa targets were inconsistently achieved [25]. These findings align with a recent meta-analysis confirming that LMWH, while safer for the fetus, may offer less reliable valve protection than VKAs when monitoring is inadequate [26].

### 3.3. Practical Management Strategies

Current ESC (2025) and AHA (2020) guidelines outline three principal anticoagulation approaches for women with mechanical valves [1,2,26]:

- VKA throughout pregnancy except near delivery—ensures maximal valve protection but carries the greatest fetal risk, particularly at higher doses.
- Sequential therapy—LMWH or UFH during the first trimester (to avoid teratogenic exposure), followed by VKA until 36 weeks, then switching back to heparin near term.
- LMWH for the entire pregnancy—used when VKA avoidance is prioritized, requiring rigorous anti-Xa monitoring (target peak 0.8–1.2 U/mL, trough > 0.6 U/mL).

Peripartum management involves discontinuing VKA at 36 weeks, withholding LMWH 24 h before planned delivery, and stopping intravenous UFH 4–6 h pre-procedure. UFH’s short half-life makes it preferable in the immediate peripartum period. After delivery, warfarin can be safely resumed and is compatible with breastfeeding [1,2].

**Figure 2:**
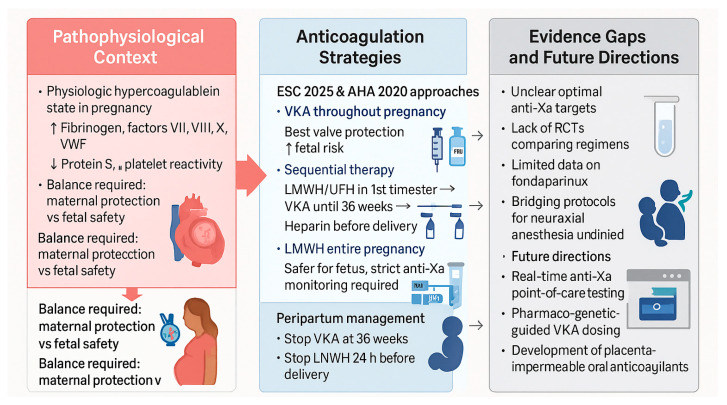
Anticoagulation strategies and management of mechanical heart valves during pregnancy. Abbreviations: AHA, American Heart Association; ESC, European Society of Cardiology; LMWH, low-molecular-weight heparin; UFH, unfractionated heparin; VKA, vitamin K antagonist; VWF, von Willebrand factor; RCT, randomized controlled trial; anti-Xa, anti–factor Xa activity.

Figure 2 summarizes anticoagulation strategies and management of mechanical heart valves during pregnancy.

Individualization is essential. Maternal thrombotic risk depends on prosthesis type (mitral > aortic), prior thrombosis, and the adequacy of anticoagulation monitoring. Women using LMWH require anti-Xa assays at least weekly, and management should occur in tertiary centers with cardiology, hematology, and obstetric expertise.

Bioprosthetic valves, while avoiding long-term anticoagulation, carry a risk of structural degeneration that may be accelerated by pregnancy. Hence, the decision between mechanical and bioprosthetic valve replacement in women of childbearing potential must balance durability against peripartum risk, ideally made before conception.

The main anticoagulation strategies for women with mechanical heart valves and their respective monitoring protocols are summarized in Figure 2.

### 3.4. Evidence Gaps and Research Needs

Several critical uncertainties persist. Optimal anti-Xa targets for LMWH remain undefined, with disagreement over whether peak or trough levels better predict thrombotic protection. No large randomized controlled trials (RCTs) have directly compared VKA and LMWH regimens, and most current evidence derives from observational registries. Furthermore, data on emerging agents such as fondaparinux are limited to case reports or small series, restricting their use to women with heparin allergy [27].

There is also a paucity of information on peripartum hemostatic management, particularly regarding bridging strategies and timing of neuraxial anesthesia. The establishment of standardized, prospective monitoring protocols could reduce both thrombotic and hemorrhagic complications.

### 3.5. Future Perspectives

Technological and pharmacological advances may improve safety in this population. Real-time anti-Xa point-of-care testing and pharmacogenetic profiling for VKA metabolism could enhance dosing precision. The development of reversible, placenta-impermeable oral anticoagulants remains a key unmet need. International registries, including ROPAC and EORP Pregnancy, continue to provide invaluable insights into global practice variability and outcomes.

The integration of risk-prediction algorithms considering prosthesis type, prior events, and anticoagulation history could facilitate individualized counselling. Additionally, the role of pre-pregnancy valve replacement strategy should be further studied: recent evidence suggests that bioprosthetic valves may offer favorable peripartum outcomes when re-intervention options (e.g., transcatheter valve-in-valve) are available.

## 4. Hypertensive Disorders of Pregnancy and Long-Term Cardiovascular Risk

### 4.1. Pathophysiology and Clinical Spectrum

Hypertensive disorders of pregnancy (HDPs), including chronic hypertension, gestational hypertension, pre-eclampsia, and eclampsia, represent the most prevalent cardiovascular complications in pregnancy and a leading cause of maternal and perinatal morbidity worldwide [28,29]. Pre-eclampsia, defined by new-onset hypertension with proteinuria or end-organ dysfunction after 20 weeks’ gestation, affects 2–8% of pregnancies and remains responsible for approximately 70,000 maternal deaths annually [30].

The pathogenesis of pre-eclampsia is complex and multifactorial, involving abnormal placentation, systemic inflammation, oxidative stress, and endothelial dysfunction. In early gestation, impaired trophoblastic invasion of the spiral arteries results in placental ischemia, which in turn triggers the release of anti-angiogenic and inflammatory mediators into the maternal circulation. An imbalance between soluble fms-like tyrosine kinase-1 (sFlt-1) and placental growth factor (PlGF) plays a central role: excess sFlt-1 neutralizes VEGF and PlGF, reducing nitric oxide bioavailability and disrupting vascular integrity [31,32]. This angiogenic imbalance contributes to generalized endothelial dysfunction, vasoconstriction, and multi-organ involvement typical of pre-eclampsia.

Beyond its immediate obstetric implications, pre-eclampsia is now recognized as a potent marker of future cardiovascular risk. Epidemiological studies demonstrate a two- to fourfold increased lifetime risk of chronic hypertension, ischemic heart disease, heart failure, and stroke among women with prior pre-eclampsia [33,34,35]. These findings suggest that pregnancy serves as an early “stress test” for cardiovascular vulnerability, unmasking pre-existing endothelial and metabolic susceptibilities. Shared pathophysiological pathways—oxidative stress, vascular inflammation, and lipid abnormalities—link HDPs with later atherosclerotic disease [36].

**Figure 3:**
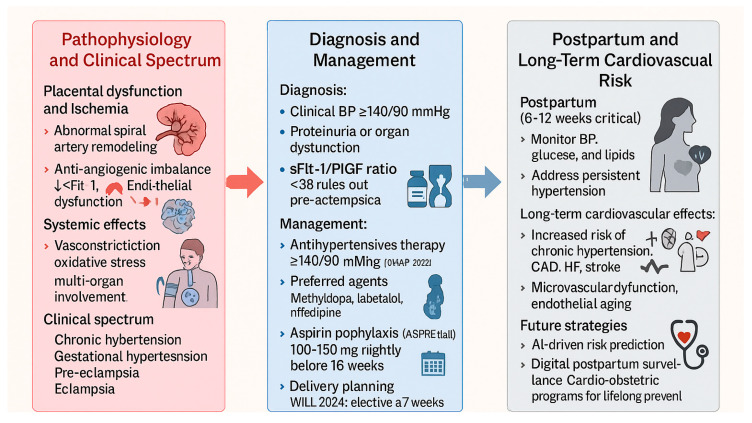
Hypertensive disorders of pregnancy: pathophysiology, management, and long-term cardiovascular risk. Abbreviations: ACEi, angiotensin-converting enzyme inhibitor; AI, artificial intelligence; ARB, angiotensin receptor blocker; BP, blood pressure; CAD, coronary artery disease; CHAP, Chronic Hypertension and Pregnancy trial; HF, heart failure; HDP, hypertensive disorders of pregnancy; MRA, mineralocorticoid receptor antagonist; PlGF, placental growth factor; sFlt-1, soluble fms-like tyrosine kinase-1; VEGF, vascular endothelial growth factor.

The multifactorial pathophysiology of hypertensive disorders of pregnancy, including abnormal placentation, angiogenic imbalance, oxidative stress, and endothelial dysfunction, is illustrated in Figure 3.

### 4.2. Diagnostic Advances and Biomarkers

Traditional diagnosis of HDPs relies on clinical criteria—blood pressure thresholds and the presence of proteinuria or organ dysfunction. However, these features often appear late in disease evolution, limiting preventive intervention. The introduction of angiogenic biomarkers, particularly the sFlt-1/PlGF ratio, has markedly improved diagnostic precision and risk stratification.

A high sFlt-1/PlGF ratio reflects anti-angiogenic predominance and correlates with disease severity, risk of preterm delivery, and adverse maternal and fetal outcomes. The PROGNOSIS study demonstrated that a ratio ≤38 effectively rules out pre-eclampsia within seven days, while elevated ratios predict imminent disease [37]. Integration of biomarker-guided algorithms into clinical practice may enable earlier detection and targeted monitoring, especially in high-risk women with chronic hypertension, renal disease, or prior pre-eclampsia [38].

Emerging technologies such as machine learning and multimodal imaging further refine risk prediction. Artificial intelligence models incorporating maternal demographics, hemodynamic indices, and angiogenic profiles outperform traditional scoring systems in predicting pre-eclampsia onset and severity [39]. Moreover, non-invasive assessment of vascular function—such as flow-mediated dilation and pulse-wave velocity—offers additional insight into persistent endothelial dysfunction postpartum [40].

### 4.3. Therapeutic Management and Comparative Guidelines

Effective management of HDPs aims to prevent maternal complications while optimizing fetal growth and prolonging gestation. Lifestyle modification, pharmacological therapy, and timely delivery remain the cornerstones of treatment.

Antihypertensive therapy should be initiated for sustained systolic blood pressure ≥ 140 mmHg or diastolic ≥ 90 mmHg, according to the 2022 CHAP trial, which demonstrated a significant reduction in adverse outcomes with a lower treatment threshold [40]. These findings have influenced recent ESC 2025 guidelines, which now recommend initiating therapy at ≥140/90 mmHg, aligning with AHA 2020 guidance [1,2]. Methyldopa, labetalol, and nifedipine are considered safe and effective first-line agents, while ACE inhibitors, ARBs, and mineralocorticoid receptor antagonists (MRA) remain contraindicated during pregnancy [1].

Aspirin prophylaxis plays a pivotal preventive role. The ASPRE trial demonstrated that low-dose aspirin (150 mg nightly from 12 to 36 weeks) reduces the incidence of preterm pre-eclampsia by 62% among high-risk women [41]. Both ESC and AHA guidelines endorse low-dose aspirin (100–150 mg) beginning before 16 weeks of gestation in women with prior pre-eclampsia, chronic hypertension, renal disease, or autoimmune disorders. Calcium supplementation (1–2 g/day) is also recommended in populations with low dietary intake to reduce the risk of pre-eclampsia [1,42].

Delivery timing must balance maternal and fetal risk. In severe pre-eclampsia, delivery remains the definitive treatment; decisions should consider gestational age, fetal condition, and maternal stability. The WILL trial (2024) supported planned delivery at 37 weeks for women with chronic or gestational hypertension, reducing maternal complications without increasing neonatal morbidity [43]. Close postpartum monitoring is essential, as hypertension may persist or worsen in the weeks after delivery due to volume redistribution and vascular tone recovery [44].

The main pharmacological options, their safety profiles, and contraindications according to current ESC and AHA guidelines are outlined in Table 1.

### 4.4. Long-Term Cardiovascular Risk

The postpartum period offers a unique window for cardiovascular risk identification and prevention. Women with a history of HDPs should undergo blood pressure, glucose, and lipid evaluation within 6–12 weeks postpartum and periodically thereafter [1]. Persistently elevated blood pressure or evidence of metabolic syndrome warrants transition to chronic cardiovascular prevention pathways.

Endothelial dysfunction and low-grade inflammation often persist months after delivery, suggesting that pre-eclampsia accelerates vascular aging rather than representing a transient obstetric complication [33,34]. The development of microalbuminuria increased carotid intima–media thickness, and coronary artery disease (CAD) or coronary microvascular dysfunction have all been described in this population [45,46]. These findings reinforce the need for long-term surveillance and lifestyle modification to mitigate subsequent cardiovascular risk.

### 4.5. Future Directions

Research priorities include identifying biomarkers that predict long-term cardiovascular sequelae and clarifying the mechanisms linking placental ischemia to chronic vascular injury. The interplay between genetics, immune activation, and metabolic dysregulation deserves further study. Large-scale prospective cohorts are needed to evaluate whether early cardiovascular interventions in women with prior HDPs—such as statin therapy or aggressive risk factor control—can mitigate future disease.

Digital health tools and artificial intelligence (AI)-based predictive models could transform postpartum surveillance, allowing remote blood pressure monitoring and personalized follow-up. Moreover, multidisciplinary postpartum “cardio-obstetric” programs should become a standard component of maternal health systems, ensuring continuity of care well beyond delivery.

## 5. Discussion

Pregnancy-related cardiovascular disease represents a complex interaction between hemodynamic stress, hormonal adaptation, and pre-existing vulnerability. Across peripartum cardiomyopathy, valvular disease, and hypertensive disorders, a unifying theme emerges: the imbalance between endothelial resilience and systemic inflammatory and oxidative stress. Pregnancy exposes this imbalance, revealing subclinical pathology that may otherwise remain silent until later in life.

### 5.1. Shared Mechanistic Pathways

**Figure 4:**
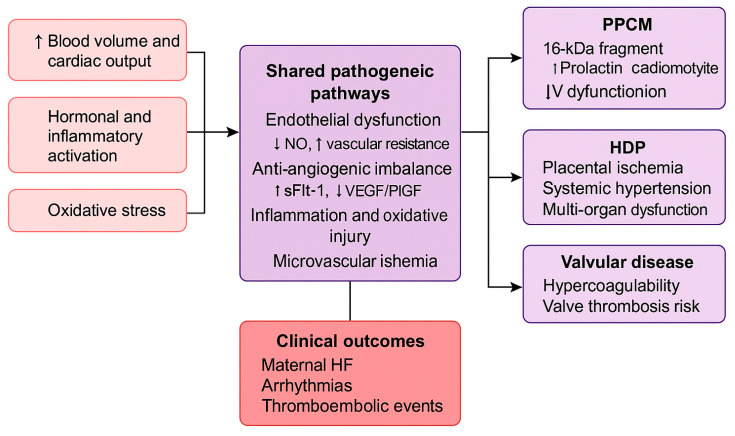
Conceptual diagram illustrating the common biological pathways underlying major cardiovascular disorders in pregnancy. Abbreviations: VEGF, vascular endothelial growth factor; PlGF, placental growth factor; sFlt-1, soluble fms-like tyrosine kinase-1; PPCM, peripartum cardiomyopathy.

A growing body of evidence suggests that diverse pregnancy-associated cardiovascular disorders share overlapping molecular mechanisms. Endothelial dysfunction is a common denominator, driven by anti-angiogenic signaling, oxidative stress, and microvascular injury. In peripartum cardiomyopathy, cleavage of prolactin into its 16 kDa fragment exerts toxic anti-angiogenic effects, while in pre-eclampsia, overexpression of sFlt-1 disrupts VEGF- and PlGF-mediated endothelial repair [14,32]. Both processes culminate in microvascular ischemia, myocardial strain, and progressive ventricular dysfunction. These shared biological mechanisms are schematically illustrated in Figure 4, which summarizes the overlapping pathogenic pathways contributing to endothelial dysfunction, angiogenic imbalance, and clinical cardiovascular manifestations during pregnancy.

Inflammation and immune activation also represent converging pathways. Elevated levels of interleukin-6, tumor necrosis factor-α, and C-reactive protein have been documented in both PPCM and pre-eclampsia, reflecting systemic endothelial activation and oxidative injury. Metabolic stress, including dyslipidemia and insulin resistance, further amplifies endothelial damage, suggesting that pregnancy acts as a biological stress test revealing latent cardiometabolic susceptibility [45,46,47].

### 5.2. Multidisciplinary Care and Lifelong Cardiovascular Health

Effective management of cardiovascular disease in pregnancy requires a structured, multidisciplinary approach integrating cardiologists, obstetricians, anesthesiologists, and neonatologists within a dedicated Pregnancy Heart Team. Both the ESC 2025 and AHA 2020 guidelines emphasize this model, underscoring coordinated care across the preconception, antepartum, and postpartum stages, though implementation remains uneven, particularly in low-resource settings [1,2].

**Figure 5:**
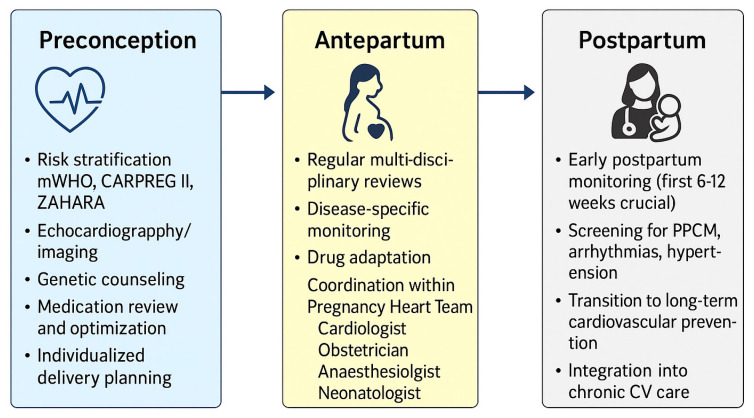
Structured multidisciplinary management of cardiovascular disease in pregnancy. Abbreviations: BP, blood pressure; CARPREG II, Cardiac Disease in Pregnancy Study II; HF, heart failure; LVEF, left ventricular ejection fraction; mWHO, modified World Health Organization classification of maternal cardiovascular risk; PPCM, peripartum cardiomyopathy; ZAHARA, Zwangerschap bij Aangeboren HARtAfwijking study.

A continuum of care beginning before conception is crucial. Preconception evaluation should include risk stratification using mWHO, CARPREG II, and ZAHARA scores, echocardiographic assessment, medication review, and individualized delivery planning. During pregnancy, regular multidisciplinary reviews enable early identification and management of complications, while postpartum surveillance focuses on recovery and long-term risk reduction. In addition to cardiac-specific disorders, several comorbid conditions significantly modulate cardiovascular risk during pregnancy. Diabetes mellitus, obesity, smoking, alcohol or drug use, and pre-existing congenital heart disease further complicate management and amplify both maternal and fetal risks. These factors highlight the need for integrated preventive strategies and tailored counselling within the Pregnancy Heart Team framework, as represented in Figure 5.

### 5.3. Clinical Evidence and Knowledge Gaps

International registries have played a pivotal role in advancing maternal cardiology. The ROPAC and EORP Pregnancy and PPCM registries have revealed considerable regional variation in management strategies, medication use, and outcomes [25,45]. Major clinical studies and registries addressing pregnancy-related cardiovascular disease are summarized in Table 2, emphasizing key findings and persistent gaps in evidence. Expanding registry participation and harmonizing data through integration with electronic health records would enhance real-world evidence and enable large-scale analyses incorporating imaging, biochemical, and genetic parameters. Furthermore, these platforms lay the groundwork for AI and machine learning applications to improve risk prediction, drug safety, and personalized management across pregnancy-related cardiovascular disorders.

Despite progress, several evidence gaps persist. In peripartum cardiomyopathy, randomized studies are required to clarify the role of bromocriptine, anticoagulation duration, and genetic determinants of recurrence. In valvular heart disease, defining optimal LMWH anti-Xa targets and monitoring strategies remains a priority. For hypertensive disorders of pregnancy, longitudinal trials should assess whether early cardiovascular interventions—such as statin therapy or intensive risk-factor modification—can prevent heart failure and atherosclerosis in later life. A concise overview of the established evidence and ongoing controversies across major pregnancy-related cardiovascular disorders is presented in Table 3.

## 6. Conclusions

Pregnancy represents a unique cardiovascular stress test, revealing latent vulnerabilities and predisposing to both acute and chronic complications. Across peripartum cardiomyopathy, valvular disease, and hypertensive disorders, shared mechanisms—endothelial dysfunction, angiogenic imbalance, and systemic inflammation—underlie the continuum between obstetric and lifelong cardiovascular health.

Integrating obstetric and cardiovascular care will transform pregnancy from a period of vulnerability into an opportunity for lifelong cardiovascular prevention.

## Figures and Tables

**Table 1:** Comparative ESC 2025 vs. AHA 2020 Recommendations in Pregnancy-Associated Cardiovascular Conditions.

Clinical Domain	ESC 2025	AHA 2020	Key Differences
PPCM	Defines PPCM as HF with LVEF < 45% developing in late pregnancy or ≤6 months postpartum. Bromocriptine recommended (Class IIa) in severe PPCM with anticoagulation.	Similar diagnostic definition. Bromocriptine may be considered in research or specialized centers.	ESC provides stronger support for bromocriptine based on registry evidence, while AHA remains cautious due to lack of RCTs.
Anticoagulation in Mechanical Valves	Individualized strategy. VKA preferred throughout pregnancy if daily dose ≤5 mg; LMWH acceptable with strict anti-Xa monitoring (peak 0.8–1.2 U/mL). Transition to heparin at 36 weeks.	Similar strategy; emphasizes shared decision-making. LMWH targets: peak 0.7–1.2 U/mL, trough monitoring optional.	General agreement, but ESC provides more specific anti-Xa targets; need for standardization persists.
Antihypertensive Therapy	Initiate at ≥140/90 mmHg (based on CHAP). Labetalol, nifedipine, and methyldopa as first-line agents.	Same BP threshold and agents.	Full alignment after CHAP; both discourage ACE inhibitors, ARBs, MRAs.
Aspirin Prophylaxis for Pre-eclampsia	100–150 mg nightly starting <16 weeks in high-risk women.	81–162 mg daily before 16 weeks in high-risk women.	Concordant strategy with minor dosing differences.
Delivery Timing in Hypertensive Pregnancy	Planned delivery at 37 weeks for stable women with chronic or gestational hypertension (WILL 2024).	Delivery between 37 and 39 weeks for controlled hypertension.	Harmonized recommendations based on recent RCTs.
Postpartum Cardiovascular Follow-up	Evaluation of BP, glucose, and lipids within 6–12 weeks; transition to cardiovascular prevention programs.	Similar recommendations emphasizing coordination with primary care.	Consensus achieved but implementation uneven across systems.

Abbreviations: AHA, American Heart Association; BP, blood pressure; CHAP, Chronic Hypertension and Pregnancy (Trial); ESC, European Society of Cardiology; HF, heart failure; LMWH, low-molecular-weight heparin; LVEF, left ventricular ejection fraction; MRA, mineralocorticoid receptor antagonist; PPCM, peripartum cardiomyopathy; RCT, randomized controlled trial; VKA, vitamin K antagonist; WILL, When to Induce Labour to Limit risk in pregnancy hypertension (Trial).

**Table 2:** Summary of Key Clinical Studies and Registries on Cardiovascular Disease in Pregnancy.

Section	Study/Registry	Population/Design	Main Findings/Clinical Message	Ref.
Peripartum and Preexisting Cardiomyopathies	EORP PPCM Registry	International prospective registry	Bromocriptine + standard HF therapy associated with higher LVEF recovery and lower 6-month mortality	[15,17]
	IPAC Study	North American multicenter cohort	~50% LV recovery at 6 months; baseline LVEF <30% predicts poor outcome	[18]
	Bromocriptine Pilot (Sliwa et al.)	Prospective interventional pilot	Prolactin blockade improves LV recovery; mechanistic support for targeted therapy	[16]
	CARPREG II	Risk prediction model	Identifies predictors of maternal cardiac events; cornerstone for pre-pregnancy counseling	[6]
	Italian Multicentre Registry (Ilardi et al.)	Multicentre national cohort	Heterogeneous clinical and echocardiographic features; LV recovery predictors require further investigation	[19]
	Arrhythmic PPCM Presentation (Peretto et al.)	Case series + critical review	PPCM may present with malignant ventricular arrhythmias; arrhythmic phenotype may precede LV dysfunction	[20]
Anticoagulation and Mechanical Valve Management	ROPAC III Registry	613 pregnancies (411 mechanical valves, 202 bioprosthetic)	Valve thrombosis 4–17%; higher risk with LMWH when anti-Xa targets unmet	[25]
	D’Souza Meta-analysis	Systematic review (VKA vs. LMWH)	VKAs provide best valve protection; LMWH safer for fetus but requires strict anti-Xa monitoring	[26]
Hypertensive Disorders of Pregnancy (HDPs)	CHAP Trial	2408 women with mild chronic hypertension	Antihypertensive treatment at ≥140/90 mmHg reduces adverse pregnancy outcomes; adopted by ESC 2025/AHA 2020	[48]
	ASPRE Trial	High-risk women, aspirin prophylaxis	Aspirin 150 mg nightly <16 weeks reduces preterm pre-eclampsia by ~60%	[41]
	WILL Trial	Chronic or gestational hypertension	Planned delivery at 37 weeks reduces maternal complications without increasing neonatal risk	[43]
	PROGNOSIS Study	Suspected pre-eclampsia	sFlt-1/PlGF ≤38 rules out pre-eclampsia for 7 days; elevated ratio predicts imminent disease	[37]
Other Pregnancy-Related CV Conditions	PPCM Registries (European cohorts)	Multicenter European cohorts	~50% LV recovery at 6 months; poor outcome associated with LVEF < 30% or LVEDD > 60 mm	[14]
	Preeclampsia and Future CVD Risk	Systematic review and meta-analysis	2–4× increased lifetime risk of chronic hypertension, IHD, and stroke after pre-eclampsia	[9]

Abbreviations: AHA, American Heart Association; CHAP, Chronic Hypertension and Pregnancy trial; ESC, European Society of Cardiology; HF, heart failure; HDP, hypertensive disorders of pregnancy; LMWH, low-molecular-weight heparin; LVEF, left ventricular ejection fraction; PE, pre-eclampsia; PlGF, placental growth factor; PPCM, peripartum cardiomyopathy; sFlt-1, soluble fms-like tyrosine kinase-1; VKA, vitamin K antagonist.

**Table 3:** Clinical Insights and Controversies across Major Pregnancy-Related Cardiovascular Disorders.

Condition	Established Evidence/Current Consensus	Unresolved or Controversial Issues
PPCM	Bromocriptine improves LV recovery when combined with standard HF therapy; anticoagulation indicated if LVEF < 35%.	Lack of randomized trials on bromocriptine; unclear recurrence risk modifiers in subsequent pregnancies.
Mechanical Valves and Anticoagulation	Low-dose VKA (<5 mg/day) remains safest for valve protection; LMWH feasible with rigorous monitoring; warfarin safe during lactation.	Optimal anti-Xa targets not standardized; limited RCT data comparing regimens; LMWH underdosing remains common.
Hypertensive Disorders of Pregnancy	CHAP and WILL trials define modern BP thresholds and delivery timing; aspirin prophylaxis reduces preterm pre-eclampsia risk by ~60%.	Need for real-world data on aspirin adherence and dosing; mechanisms linking HDP and long-term CVD still incompletely understood.
Postpartum Follow-up	HDP and PPCM recognized as markers of long-term cardiovascular risk; structured follow-up recommended at 6–12 weeks.	Implementation gaps in postpartum care; unclear optimal duration and modality of cardiovascular surveillance.

Abbreviations: BP, blood pressure; CHAP, Chronic Hypertension and Pregnancy (Trial); CVD, cardiovascular disease; HF, heart failure; HDP, hypertensive disorders of pregnancy; LMWH, low-molecular-weight heparin; LVEF, left ventricular ejection fraction; PPCM, peripartum cardiomyopathy; RCT, randomized controlled trial; VKA, vitamin K antagonist; WILL, When to Induce Labour to Limit risk in pregnancy hypertension (Trial).
